# Attenuation of osteoarthritis via blockade of the SDF-1/CXCR4 signaling pathway

**DOI:** 10.1186/ar3930

**Published:** 2012-07-31

**Authors:** Fangyuan Wei, Douglas C Moore, Yanlin Li, Ge Zhang, Xiaochun Wei, Joseph K Lee, Lei Wei

**Affiliations:** 1 Department of Orthopaedics, The Warren Alpert Medical School of Brown University/Rhode Island Hospital, 1 Hoppin Street, Providence, RI 02903, USA; 2 Department of Emergency Medicine, The First Affiliated Hospital of Kunming Medical College, 295 Xichang Road, Kunming, Yunnan, 650032, The People's Republic of China; 3 Musculoskeletal Research Laboratory, Department of Orthopaedics and Traumatology, The Chinese University of Hong Kong, 30-32 Ngan Shing Street, Shatin, Hong Kong SAR, The People's Republic of China; 4 Department of Orthopaedics, The First Affiliated Hospital of Kunming Medical College, 295 Xichang Road, Kunming, Yunnan, 650032, The People's Republic of China; 5 Ge Zhang's Lab, Institute for Advancing Translational Medicine in Bone & Joint Diseases, Hong Kong Baptist University, Kowloon Tong, Kowloon, Hong Kong SAR, The People's Republic of China; 6 Teaching Division, School of Chinese Medicine, Hong Kong Baptist University, Kowloon Tong, Kowloon, Hong Kong SAR, The People's Republic of China; 7 Department of Orthopaedics, The Second Hospital of Shanxi Medical University, Taiyuan, Shanxi, 030001, The People's Republic of China; 8 Department of Orthopaedic Surgery, Columbia University Medical Center, 630 West 168th Street, New York, NY, 10032, USA

## Abstract

**Introduction:**

This study was performed to evaluate the attenuation of osteoarthritic (OA) pathogenesis via disruption of the stromal cell-derived factor-1 (SDF-1)/C-X-C chemokine receptor type 4 (CXCR4) signaling with AMD3100 in a guinea pig OA model.

**Methods:**

OA chondrocytes and cartilage explants were incubated with SDF-1, siRNA CXCR4, or anti-CXCR4 antibody before treatment with SDF-1. Matrix metalloproteases (MMPs) mRNA and protein levels were measured with real-time polymerase chain reaction (RT-PCR) and enzyme-linked immunosorbent assay (ELISA), respectively. The 35 9-month-old male Hartley guinea pigs (0.88 kg ± 0.21 kg) were divided into three groups: AMD-treated group (*n *= 13); OA group (*n *= 11); and sham group (*n *= 11). At 3 months after treatment, knee joints, synovial fluid, and serum were collected for histologic and biochemical analysis. The severity of cartilage damage was assessed by using the modified Mankin score. The levels of SDF-1, glycosaminoglycans (GAGs), MMP-1, MMP-13, and interleukin-1 (IL-1β) were quantified with ELISA.

**Results:**

SDF-1 infiltrated cartilage and decreased proteoglycan staining. Increased glycosaminoglycans and MMP-13 activity were found in the culture media in response to SDF-1 treatment. Disrupting the interaction between SDF-1 and CXCR4 with siRNA CXCR4 or CXCR4 antibody attenuated the effect of SDF-1. Safranin-O staining revealed less cartilage damage in the AMD3100-treated animals with the lowest Mankin score compared with the control animals. The levels of SDF-1, GAG, MMP1, MMP-13, and IL-1β were much lower in the synovial fluid of the AMD3100 group than in that of control group.

**Conclusions:**

The binding of SDF-1 to CXCR4 induces OA cartilage degeneration. The catabolic processes can be disrupted by pharmacologic blockade of SDF-1/CXCR4 signaling. Together, these findings raise the possibility that disruption of the SDF-1/CXCR4 signaling can be used as a therapeutic approach to attenuate cartilage degeneration.

## Introduction

Osteoarthritis (OA) is one of the most common and disabling diseases in the elderly, affecting nearly 80% of individuals older than 75 years [1]. Current pharmacologic therapy is largely ineffective at altering progression of the disease because the mechanisms for OA remain elusive.

Chondrocytes are the only cells present in cartilage. They are responsible for the maintenance and repair of the normal extracellular matrix, and they are central to the pathophysiologic processes involved in matrix degradation during OA. The precise mechanism by which chondrocytes induce matrix degradation under osteoarthritic conditions is unclear. To this point, research has focused largely on the inflammatory cytokines, in particular interleukin-1β (IL-1β) and tumor necrosis factor-α (TNF-α) [2]. Reducing inflammatory cytokine levels with corticosteroids effectively alleviates the symptoms of osteoarthritis, but it does not prevent the progression of the disease [3-5].

Chemokines, which have been less studied in the context of osteoarthritis, are a family of small, soluble chemoattractive cytokines that direct movement of nearby responsive cells. Chemokines have also been shown to influence cell morphology, proliferation, differentiation, and other activities through the transmembrane G-protein-coupled receptors [6,7]. Of particular interest in cartilage biology is stromal cell-derived factor 1 (SDF-1), an 8-kDa chemokine originally isolated from bone marrow stromal cells [8]. SDF-1 activates a wide variety of primary cells by binding to the G-protein-coupled receptor, CXCR4 [7]. The SDF-1/CXCR4 axis is unique in that SDF-1 is the only known ligand of CXCR4 [9]. In the joint, SDF-1 is synthesized in the synovium, and CXCR4 is expressed by articular chondrocytes [10].

SDF-1 and CXCR4 play a critical role in movement of stem cells out of the bone marrow and into the circulating bloodstream [8,11,12], and SDF-1/CXCR4-knockout mice exhibit significant developmental abnormalities that lead to embryo death [13]. Interestingly, SDF-1 and CXCR4 are expressed during development in a complementary pattern in a variety of adjacent tissue pairs, which include cardiac, vascular, hematopoietic, and craniofacial tissues [9]. This complementary expression pattern suggests a paracrine regulatory mechanism whereby tissues producing SDF-1 can induce the development of the adjacent tissues that express CXCR4. A similar expression pattern has also been found in the growth-plate cartilage [14].

Recent evidence suggests that SDF-1/CXCR4 may play a role in the progression of OA. First, a dramatic increase of SDF-1 is found in the synovial fluid from the knee joints of rheumatoid arthritis and OA patients [10]. Second, *in vitro *experiments have demonstrated that SDF-1 regulates chondrocyte catabolic activity [10,15] by stimulating the release of MMP-3 and MMP-13 [7,10]. And third, synovectomy significantly reduces the serum concentrations of SDF-1, MMP-9, and MMP-13 [15]. These findings strongly suggest that SDF-1 influences cartilage matrix degeneration by stimulating the release of MMPs from chondrocytes.

This study was performed to explore further the role of SDF-1 and CXCR4 in OA pathogenesis by manipulating SDF-1 binding to CXCR4 *in vivo*. Our overall hypothesis was that disruption of SDF-1/CXCR4 signaling would reduce the release of cartilage degenerative enzymes and attenuate OA pathogenesis.

## Materials and methods

Inhibition of the SDF-1/CXCR4 signaling cascade was evaluated *in vitro *by using human cartilage explant cultures and human chondrocyte cultures, and *in vivo *by using the Duncan Hartley guinea pig model of progressive idiopathic knee osteoarthritis.

### Blockage of SDF-1/CXCR4 in human cartilage explants

#### Cartilage explant culture

The study was approved by the Institutional Review Board at Rhode Island Hospital, and informed consent was obtained from each donor. Articular cartilage samples were obtained from patients with OA at time of total knee arthroplasty (*n *= 3); two women (ages 55 and 76 years) and one man (age 55 years). At harvest, the samples were immediately placed into DMEM culture medium and transported to the laboratory, where 1.5 × 0.5-cm square full-thickness cartilage explants were cut from the normal medial tibia region (Mankin score, 0 to 2) by using scalpels and were placed into 24-well plate in DMEM culture medium containing 10% FCS (Gibco, Grand Island, NY, USA) at 37°C in 5% CO_2_.

The 1.5 × 0.5-cm explants were cut into three equal parts (0.5 × 0.5 cm) and randomly divided into three treatment groups. Explants in Group 1(*n *= 3) were incubated with SDF-1 (250 ng/ml) to evaluate the penetration of SDF-1 into cartilage. Explants in Group 2 (*n *= 3) were incubated in media containing anti-CXCR4 monoclonal antibody (100 ng/ml; R&D systems Inc., Minneapolis, MN, USA) plus SDF-1 (250 ng/ml), to evaluate the effect of receptor blockade. The explants in Group 3 (*n *= 3) were left untreated as controls. The cartilage explants and culture medium were collected on days 2 and 4.

The cultured cartilage explants from each group were rinsed with HBSS in Tissue-Tek OCT (SakuraFinetek USA, Torrance, CA, USA) and snap frozen in liquid nitrogen. Serial 10-μm sections were cut perpendicular to the cartilage surface. The sections were fixed for 20 minutes at -20°C by using 70% ethanol containing 50 m*M *glycine. Sections were treated with hyaluronidase (2 mg/ml; Sigma Chemical Co., St Louis, MO, USA) for 30 minutes at 37°C (only for SDF-1 and CXCR4 immunostaining) and permeabilized in 0.2% Triton X-100/PBS for 5 minutes at room temperature (RT). Endogenous peroxidase was quenched, and endogenous biotin and avidin binding sites were blocked by the sequential incubation with avidin and biotin for 15 minutes and a blocking solution for 10 minutes at RT.

Penetration of SDF-1 into cartilage was evaluated with immunostaining with anti-SDF-1 antibody (25 μg/ml; R&D Systems), whereas chemokine receptor CXCR4 was evaluated with anti-CXCR4 antibody (25 μg/ml; R&D Systems). Both antibodies were applied for 1 hour at 37°C, followed by incubation with biotinylated secondary antibodies for 10 minutes at room temperature. After washing with PBS, sections were incubated with a streptavidin-peroxidase conjugate for 10 minutes, followed by a solution containing diamino-benzidine (DAB; chromogen) and 0.03% hydrogen peroxide for 5 minutes. Sections were counterstained with hematoxylin. Photographs were taken with a Nikon microscope. Additional sections were stained with Safranin-O, and the severity of proteoglycan loss and cartilage damage was quantified by using the modified Mankin grading system [16].

#### Glycosaminoglycan and MMP-13 release to culture medium

Culture media were collected at the same time as the cartilage explants (days 2 and 4), and the sulfated-glycosaminoglycan was quantified spectrophotometrically by using dimethylmethylene blue dye (DMMB) with bovine chondroitin sulfate as standard controls [17]. The concentration of MMP-13 activity in the medium was quantified with ELISA (catalog no. F13M00; R&D Systems).

### Blockage of SDF-1/CXCR4 in cultured human cartilage cells

#### Chondrocyte isolation and culture

Chondrocytes were isolated from the OA cartilage samples described earlier by using our standard method [18]. In brief, small pieces of cartilage (≈0.5 g) were minced, digested with pronase (2 mg/ml, Boehringer Roche) for 30 minutes at 37°C, and then digested with bacterial collagenase (1 mg/ml; Type IA, Sigma, C 2674) for 6 to 8 hours at 37°C in a shaker. Residual multicellular aggregates were removed by filtration, and the cells were plated in DMEM containing 10% FCS, L-glutamine, and antibiotics. After cells were grown to confluence, they were split once (passage 1) and plated either in eight-well chambers (Nalge Nunc International Corp., Naperville, IL, USA) at 1 × 10^5 ^cells/well or in 100-mm-diameter culture dishes (Becton Dickinson Labware, Franklin Lakes, NJ, USA) at 1 × 10^6 ^cells/plate. At 90% confluence, the cells were cultured under serum-free conditions overnight and then treated with SDF-1 (250 ng/ml) or transfected with SiRNA CXCR4 for 4 hours before SDF-1 treatment [19].

#### Blockage of CXCR4 with siRNA

Five micrograms of plasmids containing either the pU6RNAi-CXCR4 vector [19] or the pU6RNAi empty vector [19] (gifts from Dr. Song, University of Washington), were transfected into these chondrocytes by using a high-efficiency transfection method (Human Chondrocytes Nucleofector Kit; Amaxa Inc., Gaithersburg, MD, USA). For 24 hours after transfection, the cells were incubated in media with or without SDF-1 (250 ng/ml). The total RNA and cell lysates were collected at 36 and 48 hours, respectively. Real-time RT-PCR was carried out to detect the expression of CXCR-4 and MMP-13. CXCR4 protein expression was also evaluated with Western blotting.

#### Real-time RT-PCR (qPCR)

Total RNA was isolated from chondrocytes with RNeasy isolation kit (cat. no. 74104; Qiagen USA, Valencia, CA, USA), as previously described [20]. The 1 μg of total RNA was transcribed into cDNA by using the iScripTM cDNA synthesis kit (Bio-Rad, Hercules, CA, USA), and 40 ng/μl of the resulting cDNA was used as the template to quantify the relative content of mRNA by using QuantiTect SYBR Green PCR kit (Qiagen) with DNA Engine Opticon 2 Continuous Fluorescence Detection System (MJ Research, Waltham, MA, USA). The primers were designed by using Primers Express software (BioTools Incorporated, Edmonton, AB, Canada), which yielded

CXCR-4 forward (sense) primer, AAA CTG AGA AGC ATG ACG GAC AA,

CXCR-4 reverse (antisense) primer, GCC AAC ATA GAC CAC CTT TTC AG,

MMP-13 forward (sense) primer, TGC TGC ATT CTC CTT CAG GA,

MMP-13 reverse (antisense) primer, ATG CAT CCA GGG GTC CTG GC,

18S rRNA forward (sense) primer, CGG CTA CCA CAT CCA AGG AA, and

18S rRNA reverse (antisense) primer, GCT GGA ATT ACC GCG GCT.

The 18S rRNA was amplified as the internal control. The cycle threshold values for targets genes were measured and calculated with computer software (MJ Research, Waltham, MA, USA). Relative transcript levels were calculated as × = 2^-Δ ΔCt^, in which Δ ΔCt = ΔCt E - ΔCt C, and ΔCt E = Ctexp-Ct18S, and ΔCt C = CtC-Ct18S.

#### Western blot

Total protein was extracted from cells and quantified by using the BAC Protein Assay Kit (Pierce, Rockford. IL, USA) [21]. In brief, 10 μg of total protein was electrophoresed in 10% SDS-PAGE under reducing conditions before being transferred and probed by a human anti-CXCR4 monoclonal antibody (MAB171, 1:1,000 dilution; R&D Systems) and anti-β-actin polyclonal antibody (1:1,000 dilution; Cell Signaling Technology, Danvers, MA, USA). Horseradish peroxidase-conjugated goat anti-mouse or anti-rabbit immunoglobulin G (IgG) (H+L) (1:3,000 dilution, Bio-Rad Laboratories, Richmond, CA, USA) was used as the secondary antibody. Visualization of immunoreactive proteins was achieved by using ECL Western blotting detection reagents (Amersham, Arlington Heights, IL, USA) and subsequent exposure of the membrane to Kodak X-Omat AR film.

### AMD3100 blockage of CXCR4 in the Hartley guinea pig OA model

After receipt of IACUC approval, 35 nine-month-old male Duncan-Hartley guinea pigs (0.88 kg ± 0.21 kg) were obtained from Charles River Laboratories (Wilmington, MA, USA). The animals were allocated randomly into three experimental groups: Group 1 was left untreated to serve as a primary OA control (*n *= 11); Group 2 received continuous infusion of the CXCR4 blocker AMD3100 (Mozobil; Genzyme) via osmotic minipump (*n *= 13); and Group 3 received PBS via constant infusion osmotic minipump (*n *= 11). All animals were weighed every other week and euthanized after 3 months (12 weeks) of treatment, at which point, the knees of animals were aspirated, and the hindlimbs were removed *en bloc *via careful dissection.

#### Miniosmotic pump implantation and drug delivery

The guinea pigs were anesthetized with a solution of 0.2% (vol/vol) xylazine (Rompun; Bayer Pharmaceuticals, Brussels, Belgium) and 1% (vol/vol) ketamine (Ketalar; Parke-Davis, Bornem, Belgium) in PBS. The Mini-osmotic pumps (model 2006; Alza Corporation, Mountain View, CA, USA) were inserted into small subcutaneous pockets over the dorsolateral thorax, created by blunt dissection after a small incision (~1 cm). Before insertion, the 200-μl pump reservoirs were filled with 44.44 mg/ml AMD3100 in PBS (Group 2) or PBS alone (Group 3). At an average pumping rate of 0.15 μl/per hour, each animal in Group 2 received 160 μg AMD3100 per day. Because the pumping duration of the Alzet osmotic pump was 6 weeks, the pumps were exchanged once during the course of treatment.

#### Synovial fluid collection and analysis

At death of the animals, 100 μl of isotonic saline was injected into both knees of each animal, and the knees were flexed and extended 10 times before aspiration [22]. This technique typically yielded 160 to 180 μl of saline/synovial fluid from each animal. The synovial fluid was centrifuged at 2,000 *g *for 10 minutes to remove cells and debris and then was frozen at -80°C until analysis. Five markers of articular cartilage metabolism were measured in the synovial fluid samples by following the manufacturer's instructions. SDF-1(catalog no. DSA00), pro-MMP-1 (catalog no. SMP100), and active MMP-13(catalog no. F13M00) were measured by using Quantikine ELISA kits from R&D Systems, whereas IL-1β was measured by using an IL-1β ELISA kit from Invitrogen (catalog no. KMC0011C). Colorimetric density of the developed plates was determined by using a microplate reader set to 450 nm (model BF10000; Packard Bioscience, Meridian, CT, USA). All ELISA assays were performed in duplicate. Glycosaminoglycan (GAG) concentration was measured by using a dimethylmethylene blue dye (DMMB) assay [17].

#### Blood collection and serum analysis

Blood (5 ml) was collected by cardiac puncture immediately after the animals were killed. The blood was centrifuged at 1,800 *g *for 10 minutes, and the separated serum samples were then stored at -80°C until analysis. The level of IL-1β in the serum was measured by using the same IL-1β ELISA kit and plate-reader settings used for synovial fluid testing (catalog no. KMC0011C; Invitrogen). As with the synovial fluid samples, all of the serum samples were run in duplicate.

#### Histology

On explantation, gross morphologic lesions on the tibia plateau were visualized with India-ink staining [23]. The explanted tibiae were then fixed in 10% formalin for 72 hours, followed by decalcification in 10% EDTA solution. The tibiae were hemisected on the mid-sagittal plane, and each half was embedded in a single block of Paraplast X-tra (Fisher, Santa Clara, CA, USA). Serial 6-μm-thick sections were cut at intervals of 0 μm, 100 μm, and 200 μm and collected on positively charged glass slides (Superfrost Plus; Fisher Scientific). The sections were stained with Safranin-O/fast green. Cartilage degradation was quantified by using the modified Mankin grading system [24]. Three independent and blinded observers scored each section, and the scores for all of the sections cut from the medial and lateral tibial plateaus were averaged within each joint.

### Statistical analysis

Analysis of variance (ANOVA) was used in the *in vitro *studies to compare the three groups in terms of the concentrations of GAG, MMP-13, the relative MMP-13 mRNA levels, and. in the *in vivo *studies, to evaluate the concentrations of SDF-1, GAG, pro-MMP-1, active MMP-13, and IL-1β. The weights of the guinea pigs were adjusted by using an analysis of covariance (ANCOVA), and a two-way mixed absolute intraclass correlation coefficient (ICC) for the modified Mankin score was calculated. Follow-up pair-wise comparisons between multiple experimental groups were carried out with orthogonal contrasts by using the Scheffé test (α = 0.05) and a test of homogeneity. Adjusted *P *values for the multiple comparisons were reported. Differences were considered significant at *P *< 0.05. Statistics were performed by using SPSS software (SPSS Inc.).

## Results

### SDF-1 penetrated human cartilage explants and enhanced cartilage matrix degradation

**Figure 1:**
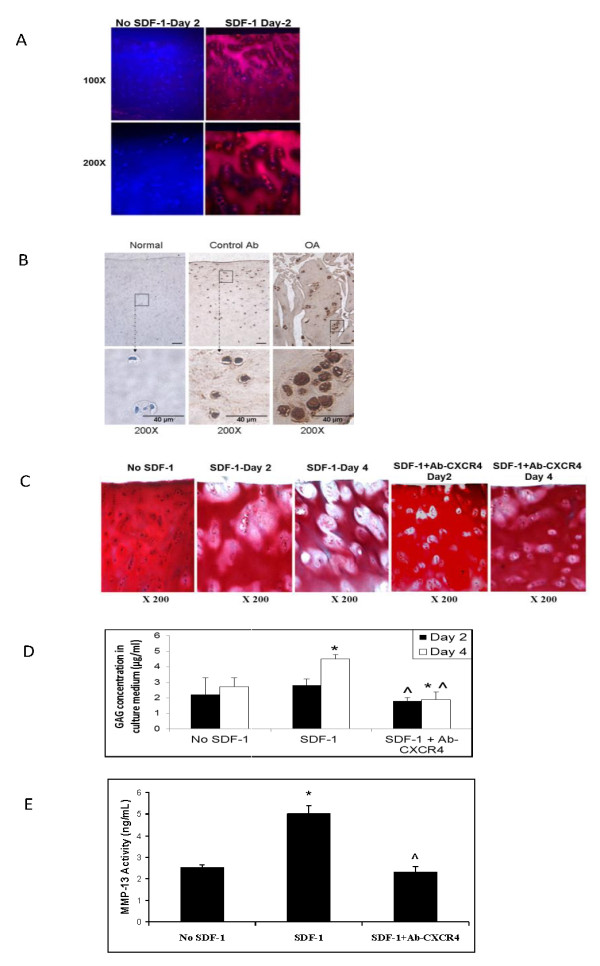
**SDF-1 infiltrated cartilage, decreased cartilage proteoglycan content, and increased both glycosaminoglycan and MMP-13 activity levels in the explants culture medium**. Immunostaining showed that SDF-1 penetrated cartilage explants partially by day 1 (top toward bottom) and completely by day 2 of SDF-1 incubation (250 ng/ml) **(A)**. Frozen sections were used to detect CXCR4 expression with immunohistochemistry. CXCR4 expression was upregulated in osteoarthritic chondrocytes (right panel, **B**) compared with normal chondrocytes (left panel, **B**). Isotype Ig antibody was used as the negative control (middle panel, **B**). Safranin-O staining of cartilage explants demonstrated proteoglycan depletion with SDF-1 treatment (**C**, panels 2 and 3), which was attenuated by pretreatment with anti-CXCR4 antibody (C, panels 4 and 5). Proteoglycan loss in the SDF-1-treated explants began in the pericellular matrix and expanded into the territorial matrix. The absence of SDF-1 incubation resulted in no loss of proteoglycan (panel 1, C). Spectrophotometry analysis demonstrated increased levels of GAG in the cultured media of explants treated with SDF-1 and no increase in levels when anti-CXCR4 antibody was preadministered (*n *= 3). *P < 0.05 compared with no SDF-1 control. ^*P *< 0.05 compared with SDF-1-treated group **(D)**. ELISA assay showed increased MMP-13 activity in the cultured media of explants treated with SDF-1 for 2 days, whereas pretreatment with anti-CXCR4 antibody before SDF-1 blocked this increase in activity (*n *= 3) (E). *P < 0.05 compared with the control. ^*P *< 0.05 compared with SDF-1-treated group.

Immunostaining revealed that SDF-1 easily penetrated human OA cartilage, with > 50% penetration after 1 day of exposure and complete penetration after 2 days (*n *= 3) (Figure 1A). Immunostaining also revealed clear expression of CXCR4 by OA chondrocytes, which was increased in osteoarthritic cartilage (*n *= 3) (Figure 1B). Inspection of Safranin-O-stained sections revealed matrix degradation after 2 days of SDF-1 incubation, with significant degenerative features after 4 days of incubation, including decreased pericellular proteoglycan content, enlarged and empty lacunae, and matrix disruption. (Figure 1C) These features were attenuated when the explants were incubated with anti-CXCR4 antibody before SDF-1 incubation (Figure 1C, two right panels). The level of GAG was significantly higher in media collected from explants treated with SDF-1 for 4 days, versus explants treated for 2 days, untreated controls or explants pretreated with anti-CXCR4 antibody before SDF-1 incubation. (Figure 1D). Finally, MMP-13 activity was increased twofold in the medium of cartilage explants treated with SDF-1, but this upregulation was suppressed completely when the SDF-1 pathway was blocked with anti-CXCR-4 antibody (Figure 1E).

### siRNA CXCR4 downregulated MMP-13 in human OA cartilage cells

**Figure 2:**
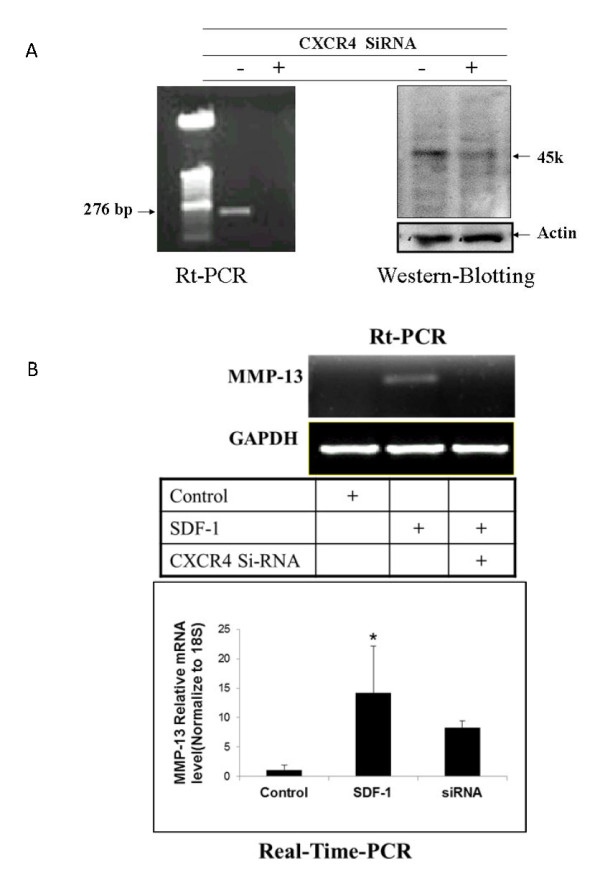
**Knockdown CXCR4 by SiRNA CXCR4 decreased MMP-13 mRNA level**. Chondrocytes were transfected with the pU6RNAi-CXCR4 siRNA or a control vector and then treated with SDF-1. Total RNA and protein were isolated for RT-PCR and Western blot, respectively. Transfection with siRNA against CXCR4 inhibited both CXCR4 mRNA and protein in comparison with the control samples without SiRNA CXCR4. **(A) **MMP-13 mRNA levels increased when SDF-1 was administered. Treatment with SiRNA CXCR4 before SDF-1 incubation eliminated the increase in MMP-13 mRNA. **(B) **Bar graphs show the averages of quantified data of MMP-13 mRNA from three independent experiments (*n *= 3). *P < 0.05 compared with control.

RT-PCR and Western blot results demonstrated that siRNA against CXCR4 inhibited both CXCR4 mRNA transcription and protein expression (Figure 2A). CXCR4 siRNA also inhibited the expression of MMP-13 (Figure 2B).

### AMD3100 attenuated the severity of OA cartilage damage *in vivo*

**Figure 3:**
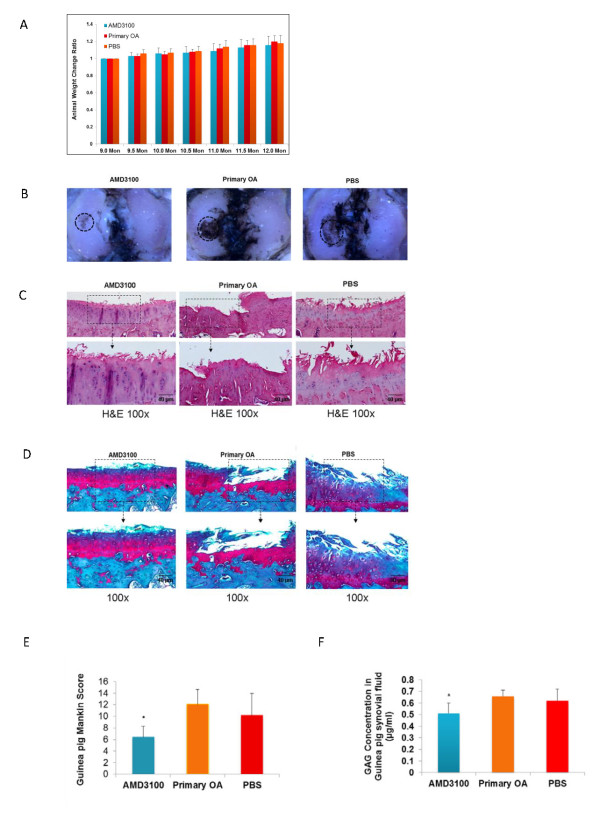
**Blocking SDF-1/CXCR4 signaling by AMD3100 attenuated the severity of OA cartilage in Hartley primary OA guinea pig model**. The weight of the animals increased gradually with time, with no significant difference among the AMD3100-treated group, 12-month primary OA group, and the sham group at any time **(A)**. India-ink stain revealed typical OA lesions in the primary OA group and PBS-treated group, whereas less staining and fewer fissures were noticed in the AMD3100-treated group **(B)**. H&E and Safranin-O staining showed less cartilage damage for the AMD3100-treated knees as compared with the 12-month primary OA and sham joints. Loss of proteoglycan staining and cartilage destruction was evident in the 12-month primary OA and sham joints **(C, D)**. Guinea pig Mankin score shows no significant difference between the primary OA group and the PBS-treated group on cartilage damage, whereas the cartilage damage in these two groups was much greater than that of the AMD3100-treated group **(E)**. The high concentration of GAG in primary OA and PBS-treated groups was attenuated in the animals treated with AMD3100 **(F)**. Data are expressed as mean ± SD. AMD3100 group, *n *= 13; primary OA group, *n *= 11; PBS group, *n *= 11. *P < 0.05.

The weight of the AMD3100-treated animals increased gradually with the time, and at roughly the same rate as that of the PBS-treated animals and untreated controls, suggesting that AMD3100 was not overtly toxic (Figure 3A). India-ink staining revealed deep and wide fissures on the central portion of the medial tibia plateaus in the animals from the OA and PBS groups, whereas in the AMD3100-treated group, the cartilage damage was much less pronounced (Figure 3B). Similarly, H&E and Safranin-O staining revealed severe OA lesions in the cartilage from both the PBS-treated group and the primary OA controls, with only minor OA changes observed in the AMD3100-treated group (Figure 3C and 3D). The Modified Mankin scores in both the primary OA and PBS control groups reflected severe degeneration (12.08 ± 2.56 and 10.18 ± 3.77, respectively; *P *= 0.308) (Figure 3E), whereas cartilage damage in the AMD3100-treated group was significantly less (6.45 ± 1.83; *P *< 0.01 for both). In the AMD 3100-treated animals, the GAG level in synovial fluid was 0.51 ± 0.09 μg/ml, which was significantly lower than that in the primary OA (0.66 ± 0.05 μg/ml; *P *= 0.006), and the PBS-treated groups (0.62 ± 0.10 μg/ml; *P *= 0.045) (Figure 3F).

### AMD3100 reduced the level of SDF-1, MMPs, and IL-1β in synovial fluid and of IL-1β in serum

AMD3100 reduced the levels of SDF-1, pro-MMP-1, active MMP-13, and IL-1β in the synovial fluid by 39%, 45%, 13%, and 45%, respectively, and reduced the serum IL-1β by 48%, in comparison to the untreated OA controls (Table 1). As expected, the synovial fluid levels of SDF-1, pro-MMP-1, active MMP-13, and IL-1β, and the level of IL-1β in serum were very similar in the PBS-treated animals and the untreated OA controls.

**Table 1:** AMD3100 reduced the levels of SDF-1, pro-MMP-1, MMP-13, and IL-1 in guinea pig synovial fluid and of IL-1 in guinea pig serum

	SDF-1, ng/ml	Pro-MMP-1, ng/ml	MMP-13, ng/ml	IL-1, ng/ml	IL-1 (serum), pg/ml
**AMD3100**	0.209 ± 0.023^a^	2.350 ± 1.101^a^	7.620 ± 0.399^a^	1.108 ± 0.416^a^	71.946 ± 40.491^a^
**Primary OA**	0.340 ± 0.071	4.286 ± 1.173	8.771 ± 0.830	2.005 ± 0.521	137.857 ± 42.998
**PBS**	0.355 ± 0.079	4.459 ± 1.764	8.382 ± 1.041	1.782 ± 0.602	156.071 ± 35.557

Data are expressed as mean ± SD. AMD3100 (*n *= 13); primary OA, *n *= 11; PBS, *n *= 11. ^a^*P <*0.05. The concentration of SDF-1, pro-MMP-1, and MMP-13 in the AMD3100-treated group was significantly lower than those of the primary OA group and the sham group. The high concentration of IL-1in synovial fluid and in serum was found in the primary OA and the PBS-treated groups, whereas blocking SDF-1/CXCR4 by AMD3100 significantly decreased the level of IL-1 in the AMD3100-treated animals.

## Discussion

Osteoarthritis (OA) is a common and disabling disease, but the mechanisms that drive the disease are unclear. Although the etiology is likely multifactorial, increasing evidence has suggested that the synovium is involved in the induction of cartilage degradation during OA and RA development [10,15]. The results of this and other studies [10,14,15,18,22] suggest that the chemokine SDF-1 plays an important role in the development of OA. A complementary expression pattern exists between SDF-1 and CXCR4; the synovium produces SDF-1, whereas its receptor, CXCR4 is preferentially expressed by articular chondrocytes [7]. Kanbe *et al. *[10] showed that human chondrocytes expressed functional chemokine receptors and released MMP-3 and MMP-13 in response to SDF-1 [10,15,25]. In organ culture, we showed that high concentrations of SDF-1 (250 ng/ml), comparable to levels observed in the synovial fluid of osteoarthritic knees (> 200 ng/ml), can readily penetrate the articular cartilage. This suggests that SDF-1 synthesized by synovial cells can diffuse freely into the adjacent cartilage. Previous studies showed that SDF-1 binds to glycosaminoglycans in the extracellular matrix or on the cell surface [26-28], which may stabilize SDF-1 and result in its accumulation around chondrocytes. Pericellular accumulation of SDF-1 around OA chondrocytes, in which CXCR4 is upregulated [15] (Figure 1B), could heighten the response to SDF-1 and induce cartilage matrix degradation.

With Safranin-O staining, we demonstrated that the cartilage explants treated with SDF-1 for as little as 2 days had significantly less proteoglycan (PG) content than did untreated explants, whereas GAG levels were higher in the media of explants treated with SDF-1. This result is consistent with the findings of previous studies in which severe OA is associated with a decrease in PG content [29,30]. Enlarged and empty lacunae were also observed in the cartilage treated with SDF-1, consistent with previous studies linking OA to decreased chondrocyte numbers and empty lacunae [31-33].

In our investigation, the cartilage-degrading enzyme MMP-13 was upregulated with SDF-1 treatment. This is consistent with previous studies, which showed that SDF-1 increases MMP-3 and MMP-13 levels in a dose-dependent manner [9,14,15,34]. We also demonstrated that the SDF-1-induced increases of MMP-13 levels can be disrupted by blocking the SDF-1 pathway with either anti-CXCR4 antibodies or siRNA directed against the CXCR4. Based on these findings, we propose that in OA, the synovium produces high levels of SDF-1 which induces matrix degradation via the release of MMP-13. Loss of the matrix disrupts the microenvironment surrounding chondrocytes, which leads to cell dysfunction and death. The cycle between matrix degradation and chondrocyte loss ultimately results in gross changes in cartilage characteristics of OA.

To test whether disruption of SDF-1 signaling could attenuate OA pathogenesis *in vivo*, we treated the primary Hartley guinea pig OA model with AMD3100, a specific nonpeptide CXCR4 chemokine receptor antagonist [35]. Dunkin Hartley guinea pigs develop spontaneous OA of the knee at around age 9 months, and the gross lesions are invariably present at 12 months [30,36]. The data of body weight indicated the animal tolerated AMD3100 well at the dose of 160 μg/per day, a dose that has been reported safe in animals and humans [37-39]. After 3 months of treatment, the gross and histology observations displayed less cartilage damage compared with the primary OA group and the sham group. The cartilage damage severity quantified by the Mankin score further indicates that AMD3100 treatment has the lowest Mankin score, almost half that of the primary OA group. Thus, the OA severity was attenuated by the injection of AMD3100.

Elevated concentrations of SDF-1 in synovial fluid have been observed in OA patients [10] and the guinea pig OA model [22]. Our data demonstrate that this pathologic elevation can be inhibited by blocking SDF-1/CXCR4 signaling with AMD3100. The regulation of SDF-1 expression is not fully understood. One study found that hypoxic conditions in the synovium of rheumatoid arthritis may induce production of SDF-1, contributing to the persistence of synovitis [40]. Other recent studies showed that interleukin-1 appears to induce SDF-1 expression in human subacromial bursa [41], and that SDF-1 expression in bursal cells can be inhibited by steroid and nonsteroidal antiinflammatory agents [42]. These studies suggest that SDF-1 levels are likely associated with the progression of inflammation. In this study, blockage of the SDF-1/CXCR4 pathway by AMD3100 reduced the level of IL-1, which indirectly reduced SDF-1 level in synovial fluid through the IL-1-SDF-1 regulation pathway.

AMD3100 also reduced the level of GAG, MMP-13, MMP-1, and IL-1β in synovial fluid and IL-1β in serum. Matrix metalloproteinases (MMPs) play an important role in the extracellular matrix degradation [43]. Previous work showed that MMPs are regulated by the SDF-1/CXCR4 axis in OA and growth-plate chondrocytes [10,14,18]. In the present study, AMD3100 inhibited MMP-1 and MMP-13 release in OA animals. Thus this association raises the intriguing possibility that AMD3100 may reduce MMPs expression through the SDF-1/CXCR4 axis. IL-1β is one of the important factors in OA pathogenesis [44] causing collagen and aggrecan breakdown [45]. Targeted reduction of IL-1β via RNAi in guinea pig chondrocytes showed beneficial effect on the OA pathogenesis [46]. In our study, the IL-1β level in synovial fluid is much higher than in serum, which is consistent with previous reports [47-49]. Our study shows that AMD3100 decreases the IL-1β levels in both synovial fluid and serum, which may reduce its harmful effects on OA progression.

Our research suggests that chemokines, along with proinflammatory cytokines, play critical roles in the pathogenesis of OA. We demonstrated that SDF-1/CXCR4 signaling directly induces cartilage matrix degradation via MMP-13 release, and that this destruction can be inhibited by blocking the SDF-1/CXCR4 pathway. The loss of matrix integrity directly compromises the mechanical properties of cartilage and may act as an accelerating force in the progression of OA. We also demonstrated that AMD3100 decreases the level of several OA-related factors in our animal model, attenuating the severity of primary OA. The age-related tissue wear results in fibrillation of the articular surface. This mechanical damage of the cartilage surface allows increasing amounts of inflammatory chemokines, such as SDF-1, to infiltrate into the cartilage. Once in the matrix, SDF-1 can then interact with CXCR4 on the cell surface, resulting in the release of MMPs and subsequent cartilage degeneration. Addition of AMD3100 blocked the binding of SDF-1 with CXCR4, removing the stimulatory trigger for the release of matrix-degrading enzymes, such as MMPs. Thus, further damage of the joint was suppressed.

## Conclusions

In summary, in this study, we demonstrated that SDF-1 directly induces cartilage matrix degradation via MMP-13 release and that the severity of OA cartilage degeneration can be attenuated by blocking SDF-1/CXCR4 signaling. Our data present a novel therapeutic target for the prevention and treatment of osteoarthritis.

## Abbreviations

ANCOVA: analysis of covariance; ANOVA: analysis of variance; DAB: diamino-benzidine; DMEM: Dulbecco Modified Eagle Medium; DMMB: dimethylmethylene blue; EDTA: ethylenediaminetetraacetic acid; ELISA: enzyme-linked immunosorbent assay; FCS: fetal calf serum; GAG: glycosaminoglycan; ICC: interclass correlation coefficient; IL-1: interleukin-1; MMP: matrix metalloprotease; OA: osteoarthritis; PBS: phosphate-buffered saline; SDF-1: stromal cell-derived factor-1; siRNA: small interfering RNA; TNF: tumor necrosis factor.

## Competing interests

The authors declare that they have no competing interests.

## Authors' contributions

FW participated in the study design, wrote most of the manuscript, performed most of the experiments, and analyzed data. DCM, GZ, YL, XW, and JKL participated in the study design, data interpretation, and revised the manuscript critically. LW conceived of the study, participated in its design, data analysis, and revised the manuscript carefully and critically. All authors read and approved the final manuscript.
